# Veganism and eating disorders: assessment and management considerations

**DOI:** 10.1192/bjb.2021.37

**Published:** 2022-04

**Authors:** Sarah J. Fuller, Andrea Brown, Jeanette Rowley, Jade Elliott-Archer

**Affiliations:** 1 East London NHS Foundation Trust, UK; 2 Schoen Clinic York, UK; 3 The Vegan Society, Birmingham, UK; 4 Irwin Mitchell LLP, Birmingham, UK

**Keywords:** Veganism, anorexia nervosa, in-patient, education and training, psychiatry and law

## Abstract

The number of people following a vegan diet in the UK is increasing. Eating disorder clinicians are anecdotally reporting that more of their patients with anorexia nervosa are wanting to follow a vegan diet. The relationship between veganism and eating disorders is unclear. A fictitious scenario is used to explore these issues. An approach is described that clinicians may follow to help patients to understand the potential relationship between their eating disorder and veganism. The human rights issues this involves are also explored. It is hoped that this article will make readers more aware of this complex issue and the impact it can have on engagement with services and on treatment options.

## Clinical scenario

You are a trainee in a tier 4 specialist eating disorders unit (SEDU) and a 22-year-old female has been admitted under section 3 of the Mental Health Act 1983 (MHA) with a body mass index (BMI) of 12.7 kg/m^2^, having lost 7 kg in 5 weeks. She has had amenorrhoea for 2 years. She was diagnosed with anorexia nervosa at age 17, requiring a 6-month admission, also under section 3 of the MHA, to a general tier 4 child and adolescent mental health unit. Since age 18, she has followed a strict vegan diet, whereas her family do not. She says her reasons for adopting this are ethical: a concern for the environment and animal welfare. Her family have questioned this, citing the timing of her illness and some lifestyle choices that conflict with veganism, such as using cosmetics containing animal products.

The SEDU has been unable to provide a full vegan diet. As a result, she is refusing to eat. For the past 72 h she has retired to bed and is only drinking water. At present, her physical health observations are stable. In the morning handover, the nursing staff request a professionals’ meeting following the unit's policy regarding refusal to eat.

Questions to consider:

1. What is the relationship between veganism and eating disorders?
2. How can clinicians assess this?
3. What are the patient's rights with regard to dietary choice on the in-patient unit?
4. What are the nutritional considerations of a vegan diet?
5. What are the implications for refeeding patients who are vegan?

## Discussion

### The complex relationship between veganism and restrictive eating disorders

The Vegan Society defines veganism as:

‘A philosophy and way of living which seeks to exclude – as far as is possible and practicable – all forms of exploitation of, and cruelty to, animals for food, clothing or any other purpose; and by extension, promotes the development and use of animal-free alternatives for the benefit of animals, humans and the environment. In dietary terms it denotes the practice of dispensing with all products derived wholly or partly from animals.’^1^

Compared with vegetarianism, veganism requires extensive dietary and lifestyle restrictions to avoid any product derived from animals.

Clinicians who support patients with restrictive eating disorders such as anorexia nervosa will be aware of the number of ways that patients can restrict their diet, ranging from total caloric restriction and self-diagnosed dietary allergies/intolerances to socially acceptable dietary restrictions such as vegetarianism or veganism. The link between vegetarianism and the development of eating disorders is well established in the literature.^2–6^ These researchers suggest that some individuals with restrictive eating disorders adopt a vegetarian diet to limit the available dietary choices or to justify a choice that is lower in calories. However, this apparent link may be due to methodological problems encountered in some of these studies. For example, eating disorder screening tools would not be able to differentiate between dietary restraint from being vegetarian or restraint driven by an eating disorder.^7,8^

For decades, diets have claimed to assist weight loss by advocating different forms of restriction. It is known that following diets can be the trigger to developing eating disorders in vulnerable individuals.^9^ A recent example of a popular diet is the ‘clean eating movement’, which advocates eating freshly prepared, unprocessed foods and recommends eating more plant-based foods. This movement is highly active on social media, with prominent influencers advocating diets, often with no qualifications to do so. If taken to an extreme, this can lead to ‘orthorexia’ . Orthorexia describes a presentation in which the person becomes pathologically obsessed with eating healthy food.^10,11^ The term, coined by American physician Steven Bratman, does not appear in any of the classifications used in psychiatry but is used in practice to describe this presentation. For some patients, the overlap between healthy eating, dieting, clean eating, orthorexia and veganism is blurred.^12^

In the past 10 years, the number of people following a vegan diet in the UK has increased and veganism is particularly common in younger age groups,^13^ females^14^ and those living in urban areas.^15^ It is estimated that ~1% of the UK population now follow a vegan diet.^16^ Clinicians may be worried that veganism shares aspects in common with restrictive eating disorders, controlled dietary exclusion and checking of food labels. Some researchers have suggested that the theoretical link between dietary restriction seen in eating disorders is mirrored in veganism.^17,18^ Furthermore, the individuals who are more likely to adopt a vegan diet have demographic overlap with those at risk of developing an eating disorder.

At present there is a lack of research into veganism in individuals who have restrictive eating disorders. Some studies have shown that those following a vegan diet are at lower risk of developing pathological eating disorders,^19^ whereas others have highlighted that former adolescent vegans may be at increased risk of extreme ‘unhealthful’ weight-control behaviours.^20^ However, adolescence is a period in which an individual starts to become independent of their family, become more socially conscious and develop their self-identity. Many young people adopt dietary patterns that are different from their families’ and therefore clinicians need to be aware of the distinction between healthy curiosity or lifestyle choices and eating-disordered behaviours.

We conducted a flash survey, via SurveyMonkey, on 1 March 2018 to identify how many patients receiving either specialist eating disorder unit (SEDU) treatment or day hospital treatment identified as vegan on admission. In total, 65 specialist services responded, representing 1008 patients with eating disorders. Within adult services, rates of veganism were reported as 46/419 (11%) in the SEDUs and 11/173 (6.4%) in the day hospitals. In child and adolescent services, the rates were 37/230 (16%) in the SEDUs and 15/186 (8.1%) in the day hospitals. These rates are higher than the national reported prevalence^16^ of 1% and this initial finding suggests that more research is needed to identify the actual prevalence within SEDUs.

### Approach to assessing vegan diets in people with eating disorders

Clinicians should be mindful of two key questions when trying to distinguish whether an individual is following a vegan diet for ethical reasons or not. First, are ethical choices seen in non-food aspects of life, such as clothes, toiletries and use of free time? Second, is there a pattern of increasing dietary restriction, such as starting off with healthy eating, then vegetarianism and finally veganism, or were ethical concerns present before the dietary restriction began?

### What are the patient's legal rights regarding dietary choice within SEDUs?

When balancing medical decision-making with human and equality rights, clinicians should be aware that veganism is classed as a non-religious belief protected under Article 9 of the European Convention on Human Rights (the right to freedom of thought, conscience and religion).^21^ Case law and guidance^22,23^ indicates that for a belief to engage Article 9 it must:

- be sincerely held
- be a belief and not an opinion/viewpoint
- concern a weighty and substantial aspect of human life and behaviour
- attain a certain level of cogency, seriousness, cohesion and importance
- be worthy of respect in a democracy, compatible with human dignity and must not conflict with the rights of others.

These criteria can be referred to when trying to understand whether a patient's vegan beliefs are distinct from their eating disorder. It is worth noting that the beliefs of an individual cannot be decided or overridden by others, and only a court can decide whether the belief complies with the criteria.

The Human Rights Act 1998 (section 6) stipulates that a public body must not act in a way that is incompatible with a Convention right. In practice this means that patients have a right to their beliefs being respected by the organisation providing care. Providing vegan food for patients who request it would therefore ensure compliance with this obligation. However, there are defences to allegations under section 6, including, for example, vegan food not being in the best interests of the patient and whether the patient is deemed to lack capacity to make important healthcare decisions.

There are two forms of discrimination, direct and indirect. Direct discrimination occurs where, contrary to section 13 of the Equality Act 2010, certain groups/people are treated differently because they hold a particular philosophical belief. Indirect discrimination can take place where, contrary to section 19 of the Act, there is an apparently neutral policy that applies to all but has the effect of disadvantaging certain groups/people (e.g. those expressing their philosophical belief in veganism). This means that if a SEDU has an inflexible catering regimen that does not allow for veganism, it leaves the unit open to legal action stating indirect discrimination. In claims arising, an objective justification for the inflexible regime will be required. Clinicians do have a potential defence to individual claims of indirect discrimination if life-saving treatments are required, for example nasogastric tube feeding, given that there is no appropriate vegan enteral feed available at present.

### What nutritional considerations need to be taken into account in vegan diets?

With appropriate expertise and planning, there no is reason why a vegan diet should not be well balanced and sufficient to meet the nutritional needs of any individual. Vegans need to ensure that they eat a wide variety of foods and find suitable plant-based alternatives for meat and dairy products. However, research suggests that there are specific nutritional vulnerabilities within a vegan diet that require particular attention or supplementation,^24^ This has led some European countries to suggest that vegans have blood tests every 3 months to monitor their nutritional status.^25^ Current guidance in the UK (https://www.nhs.uk/live-well/eat-well/vegetarian-and-vegan-diets-q-and-a/) is for vegans to take an appropriate vitamin and mineral supplement to ensure that their nutritional needs are met.

The key nutrients of concern are vitamin B_12_, vitamin D, iodine, selenium and omega-3 fats.^24^ People who have a restrictive eating disorder may not be able to eat a sufficient quantity and variety of foods and consequently they may become deficient in these and other nutrients. Close monitoring of patients’ biochemistry is therefore advised to identify whether they are deficient in any nutrients.

### What are the implications for refeeding patients who are vegan?

It is possible to refeed a patient on a vegan diet. Following a vegan diet is not an identified risk factor for the development of refeeding syndrome. However, it is important to be aware that, in some cases, like-for-like adaptations to catering menus may result in a vegan patient having to eat a larger volume of food. This may result in psychological distress as they compare their portions with those of their non-vegan peers. It may also be problematic for patients experiencing delayed gastric emptying that results in uncomfortable bloating and pain.^26,27^

Clinicians should also be aware that, if treatment is required for micronutrient deficiency (such as calcium, phosphate or magnesium) due to refeeding syndrome, some vegan alternatives may not have the equivalent nutritional value or bioavailability.^28,29^ It is well worth having discussions with the local pharmacy to ensure that supplies of such micronutrients are available and their characteristics are summarised for use in out-of-hours and other urgent situations. However, in life-threatening emergencies, treatment should be given.^30^

There is currently only one prescribable supplement drink registered as vegan friendly in the UK (AYMES ActaSolve Smoothie®), but this is not nutritionally complete and it also not suitable for enteral feeding.^31^ However, if nasogastric tube feeding is required, given the absence of any vegan enteral feeds, clinicians should be aware that many vegans will often accept foods that contain minimal amounts of animal products, for example a soya-based enteral feed in which the only ingredient that is not vegan may be a vitamin such as vitamin D. In cases where a person refuses to accept this option, and they are deemed not to have the capacity to make such a decision, legal advice should be sought for clarification and support.

## Reflections and considerations on the clinical scenario

In the clinical scenario introducing this article the patient is at high risk of refeeding syndrome and is likely to be cognitively impaired owing to starvation. There is still an option to work with her to re-establish regular eating on a fully vegan diet, which would be the least restrictive option under the MHA. Irrespective of whether the veganism is independent of her eating disorder or not, facilitating a vegan diet in line with her beliefs will result in her feeling understood and will allow the therapeutic relationship to be repaired. Any discussions about the relationship between veganism and her eating disorder can take place when she is no longer at medical risk and is able to engage cognitively.

This scenario does pose the question: do patients with eating disorders have the right to follow a vegan diet while admitted to a SEDU? Indeed, our flash survey highlighted that not all units are able to provide a vegan diet – 15/21 adult SEDUs (71.4%) and 10/13 child and adolescent SEDUs (76.9%) that responded could not – i.e. the option of following a vegan diet while receiving tier 4 treatment is not yet universal. However, the survey did not enquire into the difference between vegan diets being available versus vegan diets being offered in practice.

Veganism is becoming much more common and it is defined as a protected characteristic under the Equality Act 2010. Therefore, SEDUs need find ways to adapt to meeting vegan beliefs just as religious beliefs are accommodated. It is unlikely that a SEDU would expect a person of Jewish faith to eat pork, for example. Provision of a complete vegan diet plan incorporating all the nutrients required to avoid refeeding syndrome and promote healthy weight restoration is possible but requires the input of a specialist dietitian.

The British Dietetic Association's Mental Health Specialist Group has endorsed an internal document to help dietitians understand whether the decision to follow a vegan diet is likely to be linked to an eating disorder or is a genuine lifestyle choice that pre-existed someone's illness (this document is not yet available outside of the BDA). In some instances, veganism can help a person recovering from an eating disorder, allowing them to discover new foods and ways of cooking, change the way they perceive food and embrace the vegan subculture. For others it may be an opportunity to restrict their diet and maintain their eating disorder.

### Practical management

In the short and medium term, i.e. during this patient's admission, her veganism can be respected but also challenged in a therapeutic way, as it is not clear that her decision to follow a vegan diet is not linked to her illness. It is important to remember that being malnourished is associated with poor cognitive flexibility, so it might be more appropriate to address this once appropriate and regular nutrition is well established. At that stage, working with the unit's dietitian, it can be challenged with modifications to her meal plan and social tasks involving eating outside of the unit with family and friends. The aim would be to expand the variety of her diet while maintaining a weight at which her body is functioning and no longer experiencing any symptoms of poor nutrition and to challenge aspects of her veganism that may have been hijacked by her anorexia nervosa. In the long term, her community eating disorder team can continue to work with her and her dietary choices as is usual practice.

Treating someone with anorexia nervosa requires that the person's religion or belief is respected while at the same time ensuring that the person is not discriminated against in terms of the quality of treatment they receive. This can produce a quandary owing to the lack of vegan sip feeds and enteral feeds, which may be required under certain circumstances. In life-saving situations some patients may be prepared to accept non-vegan treatment options. In the meantime, pharmaceutical companies are being encouraged to produce vegan alternatives.

Certain situations, such as treatment under the MHA, which could include compulsory nasogastric feeding or treatment with non-vegan medication, produce ethical dilemmas. On the one hand, the therapeutic relationship with the patient is already under strain; on the other hand, treatment could be life-saving. At present, and in the absence of equivalent vegan enteral feeds and medicines, the best that can be done is to treat the patient as you would any other, while being as collaborative as possible and minimising the use of non-vegan options.

In March 2019, a consensus statement was published outlining guidance for practitioners in the UK treating vegan patients with eating disorders.^30^ This will help services to provide appropriate treatment for these individuals.

### The real people involved

The fictitious case scenario is based on the reflections of a real patient and a carer. We obtained informed consent from both to create the scenario and to publish their anonymised reflections here.

#### A patient's reflection

‘My veganism has always been respected in 20 years of [NHS and private] treatment, and even when tube feeding/supplements were required I had a product that was soya based and only had one element that was derived from animals. Wherever possible, my medication also was free from animal ingredients. My diet was limited and often “safer”, but I wanted the opportunity to challenge myself with foods I could enjoy socially within the restrictions of my illness. After 5 years in the community, I had an admission where I felt that I was detained in part due to the unit's anti-vegan policy. I gave up. Not being listened to led to a standstill in my treatment – it was “them versus me”. Veganism was the only thing stronger than my illness: I would drink a litre of oil over a teaspoon of cow's milk. I needed tube feeding and the idea of a cow's milk-based feed was difficult to accept. My body felt like a graveyard. My mental health, identity and soul were damaged and instead of fighting anorexia I was fighting the system.’

#### A carer's reflection

‘I am 100% convinced that my daughter's request to follow a vegan diet was driven by her illness. Through her whole life I had ensured that the family had a healthy and balanced diet which included treats and party food. In our house, no food was a “bad” food. Prior to being diagnosed with anorexia, she first announced that she wanted to cut out meat, then fish, then eggs. Within three months she wanted to become vegan. We embraced family treatment and had many tantrums along the way regarding her veganism. We are now in a good place and she has admitted, guiltily, that she never wanted to be vegan and her illness drove her to pursue this as a way of restricting.’

We would like to thank both the patient and the carer, who are not related, for their contribution to this paper. Both have asked to remain anonymous.

## Conclusions

We have highlighted the increasing incidence of veganism at a national level and the flash survey has suggested increased incidence within the eating disorders population. Concerns about animal welfare, environmental considerations and health impacts appear to be driving this change. There has been little research into veganism and eating disorders and more research is needed. A fictitious case has been used to explore the approach clinicians can take to support a vegan patient with an eating disorder. This included considerations on the relationship between the eating disorder and veganism, refeeding on a vegan diet and the legal implications for patients on a SEDU. The anonymous perspective of a patient and a carer highlight the multifaceted issues inherent in recovery from an eating disorder and the nuanced role veganism can plan. Wherever possible, treatments for people with eating disorders should be person centred and therefore this is an opportunity to adapt meal plans, offer appropriate supplements and engage vegan patients in their treatment.

## Data Availability

The data that support the findings of this study are available from the corresponding author, S.J.F., upon reasonable request.

## About the authors

**Sarah J. Fuller** is an advanced specialist eating disorders dietitian with East London NHS Foundation Trust, UK. **Andrea Brown** is a consultant psychiatrist and psychotherapist in eating disorders with Schoen Clinic York, UK. **Jeanette Rowley** is a legal advisor and Chair of the Vegan Society's International Rights Network, Birmingham, UK. **Jade Elliott-Archer** is a solicitor in the Medical Negligence team of Irwin Mitchell LLP, Birmingham, UK.
